# Supplementary material for: The Systems Biology Research Tool: evolvable open-source software
Source: BMC Syst Biol. 2008 Jun 29;2:55. doi: 10.1186/1752-0509-2-55 (PMC2446383; doi:10.1186/1752-0509-2-55)
Supplement: Additional file 1 — SBRT Archive. An archive of the current version of the Systems Biology Research Tool. [file 1752-0509-2-55-S1.zip › sbrt-1.4.0/doc/developers_guide/api/sbrt/shell/text/EqualsMapEntryFormatV1.html]

EqualsMapEntryFormatV1


|  |  |  |
| --- | --- | --- |
| **Overview** \| **Package** \| **Class** \| **Use** \| **Tree** \| **Deprecated** \| **Index** \| **Help** | |  |
| **PREV CLASS**   **NEXT CLASS** | **FRAMES**    **NO FRAMES**     **All Classes** |  |
| SUMMARY: NESTED \| FIELD \| CONSTR \| METHOD | DETAIL: FIELD \| CONSTR \| METHOD |  |


---


## sbrt.shell.text Class EqualsMapEntryFormatV1<K,V>

```
java.lang.Object
  sbrt.shell.text.AbstractMapEntryFormat<K,V>
      sbrt.shell.text.EqualsMapEntryFormatV1<K,V>
```

**Type Parameters:**: `K` - the key type.: `V` - the value type.

**All Implemented Interfaces:**: Format, MapEntryFormat<K,V>

---

``` public final class EqualsMapEntryFormatV1<K,V> extends AbstractMapEntryFormat<K,V> ```

This class is a concrete implementation of `MapEntryFormat`.
The string representation of map entries is:

*key = value*.

Instances of this class are immutable.

**Author:**
:   This class was written and documented by
    Jeremiah Wright while in the Wagner lab.

---

**Constructor Summary**

`EqualsMapEntryFormatV1(SimpleFormat<K> keyFormat,
SimpleFormat<V> valueFormat)`
  
          Constructs a new map format using the provided key and
value formats.


| **Method Summary** | |
| --- | --- |
| `<S extends K> java.lang.String` | `formatKey(S key)`             Returns a formatted string representation of the provided key object. |
| `<T extends V> java.lang.String` | `formatValue(T value)`             Returns a formatted string representation of the provided value object. |
| `static EqualsMapEntryFormatV1<java.lang.String,java.lang.String>` | `getBasic()`             Returns an `EqualsMapEntryFormatV1` that does not format or parse the keys or values supplied to it. |
| `SimpleFormat<K>` | `getKeyFormat()`             Returns the format used for keys. |
| `SimpleFormat<V>` | `getValueFormat()`             Returns the format used for values. |
| `K` | `parseKey(java.lang.String key)`             Parses the provided string representation of a key object. |
| `V` | `parseValue(java.lang.String value)`             Parses the provided string representation of a value object. |

**Methods inherited from class sbrt.shell.text.AbstractMapEntryFormat**

`formatPair, formatPair, getDelimiter, getLeftPadding, getRightPadding, parsePair`

**Methods inherited from class java.lang.Object**

`clone, equals, finalize, getClass, hashCode, notify, notifyAll, toString, wait, wait, wait`

**Constructor Detail**

### EqualsMapEntryFormatV1

```
public EqualsMapEntryFormatV1(SimpleFormat<K> keyFormat,
                              SimpleFormat<V> valueFormat)
```

:   Constructs a new map format using the provided key and
    value formats.

    **Parameters:**: `keyFormat` - the format to use for keys.: `valueFormat` - the format to use for values. **Throws:**: `java.lang.NullPointerException` - if either argument is `null`.


**Method Detail**

### getBasic

```
public static final EqualsMapEntryFormatV1<java.lang.String,java.lang.String> getBasic()
```

:   Returns an `EqualsMapEntryFormatV1` that does not
    format or parse the keys or values supplied to it. In other
    words, it returns an `EqualsMapEntryFormatV1` with
    the most basic possible functionality. Empty strings are not
    allowed however.

    :   **Returns:**: an `EqualsMapEntryFormatV1` that does not format or parse the keys and values supplied to it.

---


### getKeyFormat

```
public SimpleFormat<K> getKeyFormat()
```

:   Returns the format used for keys.

    :   **Returns:**: the format used for keys.

---


### getValueFormat

```
public SimpleFormat<V> getValueFormat()
```

:   Returns the format used for values.

    :   **Returns:**: the format used for values.

---


### formatKey

```
public <S extends K> java.lang.String formatKey(S key)
```

:   Returns a formatted string representation of the
    provided key object.

    :   **Type Parameters:**: `S` - a type extending the primary key type. **Parameters:**: `key` - the key object to format. **Returns:**: a formatted string representation of the provided key object.

---


### formatValue

```
public <T extends V> java.lang.String formatValue(T value)
```

:   Returns a formatted string representation of the
    provided value object.

    :   **Type Parameters:**: `T` - a type extending the primary value type. **Parameters:**: `value` - the value object to format. **Returns:**: a formatted string representation of the provided value object.

---


### parseKey

```
public K parseKey(java.lang.String key)
```

:   Parses the provided string representation of a key
    object.

    :   **Parameters:**: `key` - the string representation of a key object. **Returns:**: the key object corresponding to the provided string.

---


### parseValue

```
public V parseValue(java.lang.String value)
```

:   Parses the provided string representation of a value
    object.

    :   **Parameters:**: `value` - the string representation of a value object. **Returns:**: the value object corresponding to the provided string.


---


|  |  |  |
| --- | --- | --- |
| **Overview** \| **Package** \| **Class** \| **Use** \| **Tree** \| **Deprecated** \| **Index** \| **Help** | |  |
| **PREV CLASS**   **NEXT CLASS** | **FRAMES**    **NO FRAMES**     **All Classes** |  |
| SUMMARY: NESTED \| FIELD \| CONSTR \| METHOD | DETAIL: FIELD \| CONSTR \| METHOD |  |


---
